# Impact of Different Phytohormones on Morphology, Yield and Cannabinoid Content of *Cannabis sativa* L.

**DOI:** 10.3390/plants9060725

**Published:** 2020-06-08

**Authors:** Lisa Burgel, Jens Hartung, Daniele Schibano, Simone Graeff-Hönninger

**Affiliations:** 1 Department of Agronomy, Institute of Crop Science, Cropping Systems and Modelling, University of Hohenheim, 70599 Stuttgart, Germany; simone.graeff@uni-hohenheim.de; 2 Department of Agronomy, Institute of Crop Science, Biostatistics, University of Hohenheim, 70599 Stuttgart, Germany; jens.hartung@uni-hohenheim.de; 3 Ai Fame GmbH, 9105 Wald-Schönengrund, Switzerland; info@aifame.ch

**Keywords:** *Cannabis sativa* L., growth regulators, indoor growth, plant morphology, length of axillary branches, number of internodes, plant height, biomass yield, cannabinoids

## Abstract

The impact of exogenously applied plant growth regulators (PGR), 1-naphthalenaecetic acid (NAA), 6-benzylaminopurine (BAP), and a mixture of both (NAA/BAP-mix), was investigated in regard to plant height, length of axillary branches, number of internodes, biomass yield and cannabinoid content of three different phytocannabinoid-rich (PCR) *Cannabis* genotypes. The results showed that total plant height was significantly reduced under the application of NAA (28%), BAP (18%), and NAA/BAP-mix treated plants (15%). Axillary branch length was also significantly reduced by 58% (NAA) and 30% (NAA/BAP-mix). BAP did not significantly reduce the length of axillary branches. The number of internodes was reduced by NAA (19%), BAP (10%), and the NAA/BAP-mix (14%) compared to the untreated control. NAA application influenced the plant architecture of the tested cv. KANADA beneficially, resulting in a more compact growth habitus, while inflorescence yield (23.51 g plant^−1^) remained similar compared to the control (24.31 g plant^−1^). Inflorescence yield of v. 0.2x and cv. FED was reduced due to PGR application while cannabinoid content remained stable. Overall, the application of PGR could be used on a genotype-specific level to beneficially influence plant architecture and optimize inflorescence yield per unit area and thus cannabinoid yield, especially in the presence of space limitations under indoor cultivation.

## 1. Introduction

*Cannabis sativa* L. has a long history of cultivation for medicinal and food purposes as well as a source of textile fibers [1,2]. Five chemotypes of *Cannabis* were recognized and classified based on their cannabinoid profile and concentration: Chemotype I has a high Δ^9^-tetrahydrocannabinol/cannabidiol (THC/CBD) ratio (>1); plants with an intermediate ratio (≈1) are defined as chemotype II; fiber-type plants with a low THC/CBD ratio (<1) are defined as chemotype III; plants containing cannabigerol acid (CBGA) as their main cannabinoid are defined as chemotype IV [3], and chemotype V contains almost no cannabinoids [4,5,6,7].

Over the next few years, the cultivation of *C. sativa* is expected to rise based on adaptations to regulatory frameworks throughout the world, which may promote the constitution of new companies and exploit products obtainable from *C. sativa*. Medical cannabis was legalized in Germany in March 2017 [8]. As such, the regulatory framework comprises a policy that provides broad access to medical *Cannabis*. Today, Germany is the leading medical *Cannabis* prescriber in Europe, followed by Italy and the Netherlands [8]. The use of CBD in nutraceuticals, cosmetics, and pharmaceuticals has renewed interest in the effects of nonpsychotropic cannabinoids in particular [9,10]. Cultivation of *C. sativa* L. was banned in many states due to its psychoactive drug component Δ^9^-THC [11]. Industrial hemp genotypes, which comply with the 0.2% THC threshold set by the European Union legislation, can be cultivated without restrictions by farmers within the EU [12]. Breeding efforts for medicinal purposes focused on CBD-enhanced chemotypes, called phytocannabinoid-rich (PCR) *Cannabis*. PCR chemotypes target contents of more than 10% CBD and less than 0.2% THC, with a minimal range of variation. However, to optimize yield and the overall content of nonpsychoactive cannabinoids, a better understanding of the relationship between morphology and flower formation is necessary.

The target for high-quality *Cannabis* production includes a continuous and uniform inflorescence yield and the production of a specific cannabinoid compound [13]. Since the pharmaceutical industry requires the highest quality, it is necessary to ensure consistency in the cannabinoid profile of *Cannabis* plants and the quality of female flowers intended for such use. To optimize inflorescence yield per unit area and thus cannabinoid yield, indoor cultivation systems with minimum space requirements are needed. Outdoor cultivation seems to be economically feasible for the isolation of pure compounds or extract preparations. Indoor cultivation has advantages in terms of quality assurance and hygienic standards, allowing contamination to be eliminated and homogeneous *Cannabis* batches to be produced under controlled conditions [14]. Even though indoor systems are expensive operations, interest regarding the efficient use of available growing space is rising.

Exogenously applied growth regulators, which are chemical analogues to phytohormones, can influence the height and side-branching of plants, resulting in greater biomass and seed production [15,16]. Naturally, they occur in plants, acting as signaling compounds at low concentrations [17]. The first discovered and best understood phytohormones are auxins (IAA) and cytokinins (CK) [18], which are classified as growth promotors according to their main function [19]. IAA, synthesized in the shoot apex in young leaves and transported basipetally to the roots [20], have keys role in the maintenance of apical dominance, as well as responsibility for cell elongation. Their main effects include rooting, stimulation, and inhibition of axillary bud outgrowth [21]. CKs are involved in meristem activity regulations [22], plant shape determination, plant adjustment to side conditions, and responses to the environment [23]. IAA and CK act either antagonistically or synergistically to control developmental processes, such as the formation and maintenance of meristem [24]. *C. sativa* plants grow vertically, focusing on the growth of one dominant main shoot, with smaller side branches surrounding it, producing small buds and resulting in a low inflorescence yield. IAA from intact shoot apexes inhibit axillary branching, whereas CK, induced by removing the shoot apex, stimulates axillar branching [25]. Hence, apical dominance is regulated by IAA and CK [25]. Additionally, shoot branching depends on the genotype, growth stage, and environmental factors, including day-length, light intensity, temperature, and nutrition [26,27]. To generate a large variety of plant forms, shoot branching is a major determinant of the plant architecture, regulated by endogenous and environmental cues [23]. Synthetic compounds with similar activities to endogenous plant hormones are called “plant growth regulators” (PGR) [28]. For example, 1-naphthalenaecetic acid (NAA) and 6-benzylaminopurine (BAP) are synthetic analogues of the endogenous phytohormones IAA and CK, respectively. The effect of phytohormones on the architecture of *C. sativa* L. has not yet been studied in detail [23].

It is hypothesized that both the removal of the shoot apex and the additional exogenous application of PGR can stimulate axillary side-branching and influence the number of short side branches. The targeted plant architecture would be a compact and bushy canopy to promote optimal air circulation and light utilization. Further, shorter side branches give plants more stability during bud development. Nevertheless, the total yield cannot be rated only by the number and weight of inflorescence, therefore the content of cannabinoids is also of great interest [13].

The aim of this study was to evaluate the impact of exogenously applied NAA, BAP, and a mixture of both PGR on plant architecture of three different PCR *Cannabis* genotypes. Total plant height, axillary branch length after removal of the shoot apex, and number of internodes were investigated in detail. Furthermore, the biomass yield of inflorescence and leaves, as well as cannabinoid content, were determined.

## 2. Materials and Methods

### 2.1. Experimental Setup

A greenhouse experiment was set up to test the impact of exogenously applied plant growth regulators (PGR) on morphological characteristics of different phytocannabinoid-rich (PCR) *Cannabis* genotypes, namely cv. KANADA, cv. FED, and cv. 0.2x. The experiment was conducted at the University of Hohenheim, Germany, beginning on 28 November 2018 and finishing on 4 May 2019. Genotypes were kindly provided by the company Ai Fame, Switzerland. Genotypes were treated every fortnight with either 1-naphthaleneacetic acid (NAA) concentrated to 10 mg L^−1^, or with 6-benzylaminopurine (BAP) concentrated to 50 mg L^−1^, or a 1:5 mixture of 50% of NAA and 50% of BAP solution (NAA/BAP-mix). The experiment included a control group (control) that was sprayed with deionized water on the respective dates. NAA and BAP were both purchased from Sigma-Aldrich, St. Louis, USA. The first application took place 6 days after planting (DAP) and the last application was at 90 DAP. The applied solution amounted for each application 7.6 mL until 32 DAP and was increased to 11.0 mL for the following applications between day 32 and day 81. On the application days 88 and 90 DAP, the applied solution was increased to 27.0 mL for each application to spray the whole aboveground biomass of the plant.

Genotypes and treatments were randomly allocated to 36 plants according to a row–column design, which was established with four rows and three columns per replicate. Thus, three complete replicates existed.

### 2.2. Synthetic Growth Regulator Preparation

For the PGR solutions, 30 mg NAA was dissolved in 3 L of distilled water and 150 mg of BAP was dissolved in 3 L distilled water, with 15 mL Tween added to each solution as a surfactant. The NAA/BAP-mixture (NAA/BAP-mix) was prepared using 50% NAA solution and 50% BAP solution. NAA, BAP, and the NAA/BAP-mix were applied by evenly spraying all leaves of the plants. The control group was treated with the same amount of deionized water to simulate the spraying effect on the leaf surface. No Tween was added to the control water.

### 2.3. Plant Material

The experimental plants were generated by vegetative propagation by cutting only the apical tips of standardized mother plants. The cuttings were dipped into a rooting hormone (0.25% 4-(3-Indolyl)-butyric acid) and gently cultivated in 25 mm × 25 mm slabs, filled up with a growing media mixture of 50% seedling substrate (Klasmann-Deilmann GmbH, Geeste, Germany) and 50% sand. The clones were sprayed with water four times a day to reach a relative humidity above 90%. After 14 days and adequate root growth, the cuttings were transplanted in seedling substrate (Klasmann-Deilmann GmbH, Geeste, Germany) into a pot that was 90 mm in diameter. The shoot apexes were removed to 9 internodes for genotype KANADA and 0.2x-genetic and 11 internodes for the auto-flowering genotype FED. The experimental plants were transferred at 15 DAP into 13 × 13 cm square pots, at 50 DAP into 18 × 18 cm square pots, and finally at 74 DAP into 10 L containers, in a growing media mixture consisting of 15% black peat, 20% fraction 1, 25% milled peat, 20% GF medium, 10% pine bark, 10% leca, 1 kg m^−3^ horn chips, and 1 kg m^−3^ NPK 12-14-24 (Klasmann-Deilmann GmbH, Geeste, Germany). Under an indoor vegetative life cycle of 18 h, sunlight was supplemented with artificial lightning using Gavita high-pressure sodium (HPS) lamps, i.e., E-Series DE FLEX EU Lamp (750 W, 400 V, 1500 μmol s^−1^), Aalsmeer, Netherlands. The experiment was irrigated by a drip irrigation system and fertilized four days a week with 0.2% of Plantaactiv 18-12-18 Type A during the vegetative growth cycle and with 0.2% of Plantaactive 10-20-30 Type B during the generative growth cycle, which was purchased from Hauert (Grossaffoltern, Switzerland). The temperature during the vegetative growth stage varied from 23.7 °C to 27.6 °C. Relative humidity varied between 22.4% and 47.5%. At 129 DAP, the experimental plants were moved into a climate chamber to a 12-h photoperiod to initiate floral development. The temperature during the generative growth cycle varied from 17.9 °C to 24.1 °C. Relative humidity varied between 30.8% and 89.2%.

### 2.4. Measurements

Measurements took place for every plant each seventh day for a total period of 132 days. Plants were measured for their total height and length of axillary branches. Depending on the genotype, 9 (for KANADA and 0.2x-genetic) or 11 (for FED) branches were measured. Nodes of each axillary branch were counted for the KANADA and 0.2x-genetic genotypes. The autoflowering characteristics and dense foliage of genotype FED made it impossible to count axillar branch nodes.

### 2.5. Plant Samples

Genotypes were harvested when 70% of the pistils had darkened. Gland heads of trichomes are clear or slightly amber at the beginning of the growth cycle. Prior to harvest, when cannabinoid levels reach their maximum, they turn cloudy. The state of trichomes was monitored with binoculars. Genotype FED was harvested at 137 DAP. Harvest of genotype KANADA and 0.2x-genetic took place at 142 and 156 DAP, respectively. Inflorescence and leaves were dried at a temperature of 20 °C for 14 days. After the drying process, dry matter was weighed and recorded in gram per single plant to determine the dry weight (DW). Subsequently, the dried plant material was ground with an ultracentrifugal mill (Retsch, Type ZM 200, Haan, Germany) to acquire a homogeneous powder, with a particle size of 1 mm. The residual moisture of each samples was measured with a moisture analyzer (DBS 60-3 of Kern and Sohn GmbH, Balingen, Germany).

### 2.6. Extraction and Quantification of Cannabinoids by HPLC Analysis

Quantitative analysis of cannabinoids, particularly cannabidiol (CBD), cannabidiolic acid (CBDA), Δ^9^-tetrahydrocannabinol (THC), and Δ^9^-tetrahydrocannabinolic acid (THCA), was performed, according to Lehmann and Brenneisen [29] with slight modifications after Burgel et al. [30]. For the cannabinoid extraction, 100 ± 10 mg of the grinded sample was dissolved in 100 mL methanol 90%/chloroform 10% (v/v) (9 + 1) composite.

An external calibration of cannabinoid quantification was performed according to Burgel et al. [30], using one standard (CAN1) containing the target compounds (CAN1: THC 2%, CBD 2%, THCA 10%, CBDA 10%). The reference cannabinoids, CBD, THC, and THCA, were purchased from Lipomed (Arlesheim, Switzerland), and was purchased from CBDA from Echo Pharmaceuticals BV (Weesp, The Netherlands).

Data were processed using ChemStation Software for LC Rev. B.04.03-SP2 (Agilent, Santa Clara, CA, USA). The retention time of the respective chromatographic target peak, was compared with the chromatographic peak of the reference to carry out a quantitative analysis. The UV spectra was used to preliminarily allocate the chromatographic peak to the reference spectra visually. The identity of the target cannabinoid was proven if the deviation of the retention time of the chromatographic peak was ≤0.5 min and the optical spectra comparison did not show any difference.

To calculate the respective cannabinoid content $C_{\mathrm{TS}}$ in mass percent [${\%}_{m/m}$], Equation (1) was used, where $A_{\mathrm{TS}}$ is defined as the peak area of the standard analyst, $B_{\mathrm{TS}}$ is defined as the peak area of the sample analyst in μV × s, V is defined as the volume, $EW_{TS_{hijkl}}$ as the weight portion of the product in mg, and $F_{\mathrm{ijkl}}$ as the residual moisture of the product in %_m/m_. Indices are defined for the *i*-th genotype in the *j*-th row, the *h*-th column of the *k*-th replicate, and the *l*-th treatment; thus, the calculation was performed for each plant.

(1) $$C_{TS_{hijkl}}\left[{\%}_{m/m}\right]=\frac{\left(A_{\mathrm{TS}}\left[\mu V\;\times \;s\right]\right)}{\left(B_{TS_{hijkl}}\left[\mu V\;\times \;s\right]/100\left[\mu L\;mL^{-1}\right]\right)}\;*\;\frac{V\left[\mathrm{mL}\right]}{EW_{TS_{hijkl}}\;\left[\mathrm{mg}\right]}\;\times \;100\;\times \;F_{\mathrm{hijkl}}\left[{\%}_{m/m}\right],$$

### 2.7. Statistical Analysis

A mixed model approach was used to analyze all traits, which were determined by the measurement of single plants. Thus, the dry weight of the leaves and inflorescence, estimation, and statistical interference of the cannabinoids present in the dried plant material were analyzed by

(2) $$y_{\mathrm{hijkl}}=\;\mu +b_{k}+r_{\mathrm{jk}}+c_{\mathrm{hk}}+\delta_{i}+\tau_{l}+{\left(\delta \tau \right)}_{\mathrm{il}}+e_{\mathrm{hijkl}},$$

where $y_{\mathrm{hijkl}}$ is the observation of the *i*-th genotype in the *j*-th row, the *h*-th column of the *k*-th replicate, and the *l*-th treatment, *μ* is the intercept, $\delta_{i}$ is the fixed effect of the *i*-th genotype,$\tau_{l}$ is the fixed effect of the *l*-th treatment, ${\left(\delta \tau \right)}_{il}$ is the fixed interaction effect of the corresponding main effects, $b_{k}$ is the fixed effect of the *k*-th replicate, $e_{\mathrm{hijkl}}$ is the plant or error effect associated with observation $y_{\mathrm{hijkl}}$, and $r_{\mathrm{jk}}$ and $c_{\mathrm{hk}}$ are the random row and column effects within the *k*-th replicate, respectively. Normal distribution and homogeneous variance of residuals were checked graphically via residual plots. If needed, the data were logarithmically transformed to fulfill the requirement concerning homogeneous variance and normal distribution. In this case, estimates were back transformed for presentation purposes only. Standard errors were back transformed using the delta method.

Total plant height was measured weekly for 20 weeks. The number of internodes and the length of axillar branches were measured weekly for 14 weeks. Thus, repeated measures were taken and Model (2) was extended by the factor measurement with 20 or 14 levels, as follows:

(3) $$y_{\mathrm{hijklm}}=\mu +t_{m}+b_{\mathrm{km}}+r_{\mathrm{jkm}}+c_{\mathrm{hkm}}+\delta_{i}+{\left(\delta t\right)}_{im}+\tau_{l}+{\left(t\tau \right)}_{ml}+{\left(\delta \tau \right)}_{il}+{\left(\delta \tau t\right)}_{ilm}+e_{\mathrm{hijklm}},$$

where $t_{m}$ is the effect of the *m*-th measurement and all other effects are defined analogous to Model (2) for each measurement *m*. As repeated measures from each row, column, and plant were taken, a first-order autoregressive variance–covariance structure with heterogeneous variance was assumed for these random effects, allowing for a serial correlation between observations taken from the same row, column, or plant. The variance–covariance structure was simplified to a homogeneous variance first-order autoregressive structure if this decreased the AIC [31], thus resulting in a better model fit. After finding significant differences via the global F-test, Fishers LSD test was performed for multiple comparisons. A letter display was used to present the results of the multiple comparisons [32]. All statistical analyses were conducted using the statistical software SAS version 9.4 (SAS Institute, Cary, NC, United States). Figures were also generated using the statistical software SAS version 9.4 and Excel 2013 (Microsoft Corporation, Washington, DC, USA).

## 3. Results

### 3.1. Plant Height

**Figure 1:**
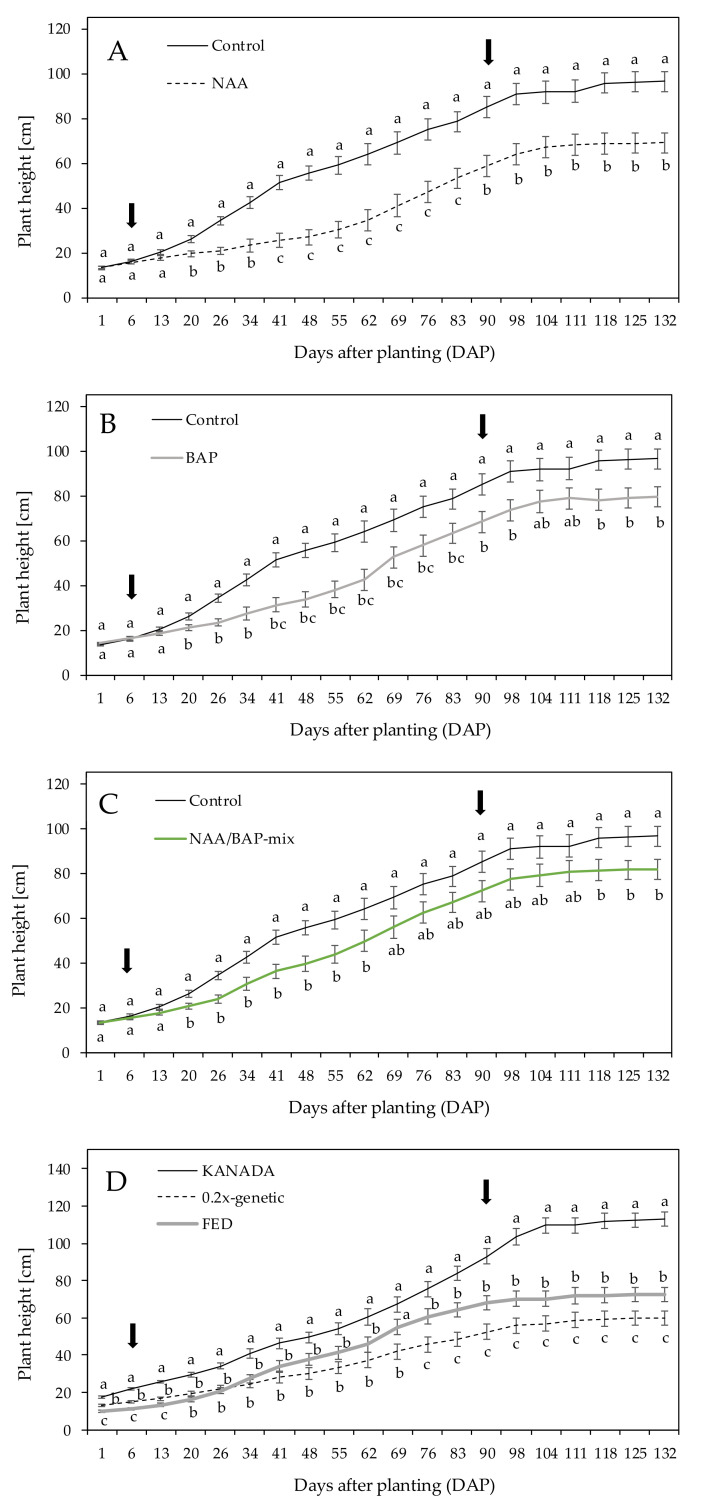
Mean plant height over all three tested genotypes treated with (**A**) 1-naphthalenacetic acid (NAA), (**B**) 6-benzylaminopurine (BAP), and (**C**) NAA/BAP-mix, compared to the nontreated control over a time period of 132 days after planting (DAP). (**D**) Mean plant height of genotype KANADA, 0.2x-genetic, and FED over 132 days. Means covered with at least one identical lowercase letter did not differ significantly at α = 0.05. The arrows show the period (6 to 90 DAP) during which application took place.

In the following section, plant height is described for different plant growth regulator (PGR) treatments (NAA, BAP, and NAA/BAP-mix) separately in comparison to control plants. The height of the treated plants showed significant interactions between treatments and measurements over time. No significant interactions were observed between treatment and genotype. Plant heights of NAA-treated and BAP-treated plants were significantly reduced compared to the control plants, starting 13 and 20 days after planting (DAP), respectively. At 132 DAP, NAA- and BAP-treated plants indicated heights of 69.20 ± 4.43 cm and 79.53 ± 4.43 cm, respectively, compared to the control with 96.47 ± 4.43 cm (Figure 1A,B). Plants treated with the NAA/BAP-mix showed the same trend (81.74 ± 4.43 cm). A significant reduction in plant height was observed between 20 DAP and 62 DAP compared to the control. No significant growth reduction was determined between 69 and 104 DAP (Figure 1C). Finally, the treatments resulted in 28% (NAA), 18% (BAP), and 15% (NAA/BAP) shorter plants in comparison to the control (Figure 1A–C).

Across all treatments, the KANADA genotype significantly indicated the highest plants at 132 DAP (113.01 ± 3.83 cm), followed by genotype FED (72.45 ± 3.83 cm), whereas genotype 0.2x-genetic indicated the shortest plants, with a final plant height of 59.88 ± 3.83 cm (Figure 1D).

### 3.2. Number of Internodes of Axillar Branches

**Figure 2:**
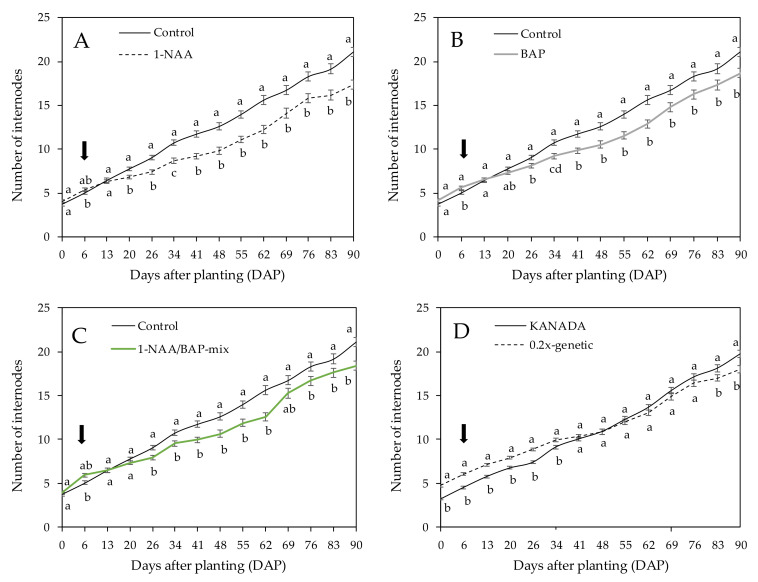
Mean number of internodes per axillary branch over two tested genotypes (KANADA and 0.2x-genetic) treated with (**A**) NAA, (**B**) BAP, and (**C**) NAA/BAP-mix, compared to the nontreated control over a time period of 90 days. (**D**) Mean number of internodes per axillary branch of genotype KANADA and 0.2x-genetic across time. Means covered with at least one identical lowercase letter did not differ significantly at α = 0.05. The arrow shows the time point (6 DAP) of the first application.

PGR treatments significantly influenced the number of internodes of axillary branches of the genotypes (KANADA and 0.2x-genetic). Internodes of the autoflowering genotype FED could not be measured. Axillary branches of NAA-treated plants showed an inhibited number of internodes after 14 days of application (20 DAP) and resulted in an average number of 17 internodes per axillary branch compared to the control, which showed 21 internodes per branch (Figure 2A). The same trend was observed for the BAP-treated and NAA/BAP-mix-treated plants. BAP and NAA/BAP-mix did not affect the number of internodes per axillary branch during the first 20 days of application (26 DAP). Between 26 and 90 DAP, a reduction of internodes was observed and resulted in 19 and 18 internodes per axillary branch compared to the control (Figure 2B,C). For the final measurement, all PGR were shown to inhibit the average number of internodes during the vegetative period compared to the control. No significant differences were observed between the treatments (Figure 2A–C).

Across all treatments, genotype KANADA showed an inhibited number of nine internodes until 34 DAP compared to genotype 0.2x-genetic, which showed inhibition of 10 internodes. Finally, at 90 DAP, genotype KANADA demonstrated a significantly higher number of internodes per axillary branch (20 internodes) compared to genotype 0.2x-genetic, which showed 18 internodes (Figure 2D).

### 3.3. Length of Axillary Branches

**Figure 3:**
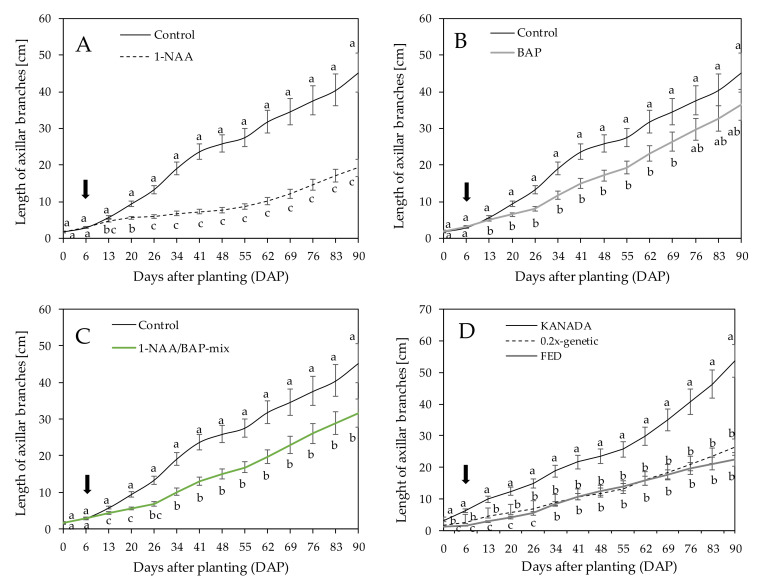
Median length of axillar branches in cm over all three tested genotypes treated with (**A**) NAA, (**B**) BAP, and (**C**) NAA/BAP-mix compared to the nontreated control each over a time period of 90 days. (**D**) Mean length of axillar branches of genotype KANADA and 0.2x-genetic across time. Means covered with at least one identical lowercase letter did not differ significantly at α = 0.05. The arrow shows the time point (6 DAP) of the first application.

PGR treatments significantly influenced the length of axillary branching. NAA-treated plants started after seven days of application (13 DAP) to inhibit the growth of axillary branches compared to the control. After 84 days of application (90 DAP), the axillar branching growth was reduced and resulted in an average length of 19.23 ± 2.25 cm (Figure 3A). After seven days of application (13 DAP), BAP-treated plants showed a significant reduction in axillar branching over the vegetative period and resulted in 36.46 ± 4.21 cm at 90 DAP compared to the control (45.26 ± 5.41 cm) (Figure 3B). A similar trend was observed for plants treated with the NAA/BAP-mix, with a final length of axillary branches of 31.63 ± 3.77 cm compared to the control (Figure 3C). At 26 DAP and 34 DAP, NAA-treated plants were shown to be influenced to a greater extent compared to the BAP-treated and NAA/BAP-mix-treated plants, respectively. NAA resulted in the highest axillary branch length reduction with 26.03 cm, while BAP and the NAA/BAP-mix showed 8.80 cm and 13.63 cm length reductions compared to the control, which was a significant finding (Figure 3A–C).

A genotype-specific difference in axillary branch length across all treatments was observed. Genotype KANADA indicated a final length of axillary branches of 53.64 ± 5.29 cm, which were the longest axillary branches compared to genotype 0.2x-genetic (26.22 ± 2.59 cm) and FED (22.46 ± 2.06 cm) at 90 DAP (Figure 3D).

### 3.4. Yield Parameters

Dry weight (DW) yield of inflorescence per plant showed significant interactions between genotype and treatment. Accordingly, DW means of the different treatments are described separately for each genotype. Control plants of genotype KANADA reached the highest inflorescence yield with 24.31 g plant^−1^, followed by plants treated with NAA (23.51 g plant^−1^) and BAP (22.97 g plant^−1^). Plants treated with a mixture of NAA and BAP (NAA/BAP-mix) indicated the lowest DW yield of inflorescence, with 14.79 g plant^−1^ compared to the control (Table 1). Control plants of 0.2x-genetic indicated the highest inflorescence yield with 20.83 g plant^−1^, followed by plants treated with BAP (15.88 g plant^−1^), which showed no significant difference compared to NAA-treated and NAA/BAP-mix-treated plants, with results of 7.61 g plant^−1^ and 8.57 g plant^−1^, respectively (Table 1). Control plants of the autoflowering genotype FED showed the highest inflorescence yield. with 32.10 g plant^−1^, which was a significant finding. NAA-treated plants indicated the lowest yield (5.39 g plant^−1^) compared to the control (Table 1).

DW yield of leaves per plant showed significant differences between treatments and genotypes. On average, genotype KANADA showed the highest DW yield of leaves (25.14 g plant^−1^) over the treatments compared to 0.2x-genetic (18.54 g plant^−1^) and FED (17.76 g plant^−1^; Table 2), which was a significant finding.

Plants treated with NAA indicated the lowest DW yield (12.43 g plant^−1^) of leaves compared to the control and the other treatments over the three genotypes, ranging from 25.60 g plant^−1^ (control) to 20.79 g plant^−1^ (NAA/BAP-mix; Table 2), which was a significant finding.

### 3.5. Cannabinoid Content

Cannabinoid acids occur in planta and undergo decarboxylation to their neutral forms upon heating or pyrolysis. The following results for cannabidiol (CBD) refer to the sum of cannabidiolic acid (CBDA) and CBD analyzed. Inflorescence and plant leaves treated with NAA, BAP, or NAA/BAP-mix did not show any statistical differences between the plants exposed to growth regulators and the control plants. Significant differences in CBD content were only between genotypes. KANADA indicated the highest content of CBD in inflorescence (10.33%) and leaves (7.03%), followed by the inflorescence (7.91%) and leaves (6.77%) of 0.2x-genetic. The lowest content was measured in the inflorescence and leaves of the autoflowering genotype FED, with 6.34% and 5.59%, respectively (Table 3).

## 4. Discussion

Exogenous application of plant growth regulators (PGR; 1-naphthaleneacetic acid (NAA), 6-benzylaminopurine (BAP), and a mixture of both (NAA/BAP-mix)) was shown to have an impact on modifying the plant architecture of *C. sativa*.

Auxins (IAA) promote stem elongation and maintain apical dominance, including inhibition of axillary bud outgrowth [21]. Hence, an increase in plant height, which leads to taller plants on average, through exogenous NAA treatment was expected. However, the results showed reduced plant height. Between 41 to 83 DAP, a reduction in plant growth in NAA-treated plants was larger compared to NAA/BAP-mix-treated plants. Mendel et al. [23] reported a nonsignificant inhibition of the average plant height of *C. sativa* over a time period of 56 days, in which plants were exposed to different NAA concentrations (5 mg L^−1^, 10 mg L^−1^, and 20 mg L^−1^). In contrast, Lalge et al. [33], documented an increase in total plant height of *C. sativa* plants treated with 10 mg L^−1^ NAA, whereas concentrations of 5 mg L^−1^ and 20 mg L^−1^ showed no significant effect on plant height [33]. Our study indicated that NAA-treated plants showed the highest axillary side-branch reduction, with an average length of 19.23 cm, compared to BAP treated plants (36.46 cm), NAA/BAP-mix treated plants (31.36 cm), and control plants (45.26 cm). However, this was in accordance with the measured number of internodes per axillary side branch, which indicated 19% (four internodes) reduction in the number of internodes per axillary side branch after NAA application compared to the control, whereas no significant differences between treatments were observed. Lalge et al. [33] and Mendel et al. [23] indicated increased lateral branching of plants subjected to NAA treatment at all concentrations that affected the plant. Lalge et al. [33] argued that buds of *C. sativa* demonstrate decreased sensitivity to the inhibitory effects of IAA in apical dominance. Since the apical dominance in our study was broken before application, this may explain the different response of the plant to NAA application, whereas Mendel et al. [23] explained the inhibitory effect in terms of plant height and the significant promoting effect in terms of axillary side-branch length by an over-optimal NAA concentration and an imbalance in the hormonal endogenous balance. Other studies showed that high IAA concentrations induced ethylene synthesis. The release of this gas could be responsible for the decrease in stem elongation [34]. Further, synthetic auxin (NAA) in *C. sativa* could interact with a different set of receptors than the most common and natural auxin indole-3-acetic acid (IAA) [23]. In the case of plant height and axillary side-branch growth, even low concentrations (5 mg L^−1^) were sufficient to achieve active stem elongation. Higher concentrations of NAA (10 mg L^−1^ and 20 mg L^−1^) eliminated and restored the hormonal effect. This biphasic behavior of the reaction could depend on adjustment of the ratio, with concentrations of exogenous auxins exceeding the minimum effective dose [23].

PGR, especially growth retardants including CKs, are generally applied in order to obtain short and compact plants [35]. CKs are involved in meristem activity regulation [22], stem elongation inhibition, and plant flowering, among other things [35]. In apical dominance, CK has an antagonistic effect to the IAA effects of. Studies of other species showed increased CK levels (25-fold within 24 h) after decapitation of the shoot apex [36]. Therefore, axillary bud outgrowth is correlated with CK concentration, which is locally biosynthesized in the nodal stem [37]. Further, it is well known that direct application of CKs to axillary buds promotes their outgrowth, even in intact plants [38]. The aim of exogenous CK application is to obtain well-branched plants without removing the apical meristem. The removal of the apical shoot apex is a common pruning technique to control the way plants grow, either to restrict plant height, maximize yield [39], or as a management tool to optimize space utilization for indoor cultivation.

Decapitation of the shoot apex to reach a defined number of side branches, in combination with exogenously applied synthetic analogues of CK (BAP), met expectations and resulted in an 18% reduction in total plant height after 90 days of application in comparison to control plants. The average length of axillary side branches showed a reduction trend of around 20% compared to the control. However, no significant reduction in axillary side branches was shown. The measured number of internodes of the axillary side branches was reduced by 9% on average compared to the control. In contrast, Mendel et al. [23] and Lalge et al. [33] reported that treatments with BAP did not affect the total plant height of *C. sativa* at concentrations of 10 mg L^−1^, 25 mg L^−1^, and 50 mg L^−1^. A total plant height of 200 to 220 cm was documented by Lalge et al. [33] 56 days after the first application took place for all concentrations, including the control, in accordance with a study by Leite et al. [40], where exogenously applied CK was not effective in modifying evaluated plant growth parameters of other plant species (*Glycine max* L. Merr.). Further, a strongly significant stimulation of axillary side-branch growth was documented at the highest BAP concentration (50 mg L^−1^). Control plants measured 1 cm in side-branch length on average, whereas BAP-treated plants measured 10 cm in side-branch length on average 56 days after the first treatment took place [23]. Lalge et al. [33] stated a concentration-dependent increase in axillary branch length of BAP-treated plants. The highest dosage (50 mg L^−1^) showed the most significant results, with around 50% increase in average axillary side-branch length compared to the control, 56 days after first application. These findings were in contrast to the present results, which can be explained by a genotype-specific reaction of PGR. While Mendel et al. [23] and Lalge et al. [33] used an industrial hemp fiber variety called Bialobrzeskie, of chemotype III, a phytocannabinoid-rich (PCR) chemotype was used in the present study. Further, by removing the shoot apex, a change in IAA/CK balance was expected.

A similar trend applied to plants treated with a mixture of both (NAA/BAP-mix). In line with NAA and BAP treatments, a reduction (15%) in total plant height after 84 days of application (90 DAP) was measured compared to the control. The length of axillary side branches was reduced by 31% on average, with the number of internodes reduced by 14% compared to nontreated control plants. IAA and CK interact in a complex manner to control plant growth [41]. Experiments with the model plant *Arabidopsis thaliana* showed a homeostatic regulatory feedback loop model, in which CK functioned as a positive regulator of IAA biosynthesis and IAA repressed CK biosynthesis [41,42,43]. It remains unclear whether this model can be extrapolated to the functioning of the whole plant, in which IAA and CK concentrations are modified naturally or in response to exogenous application [44]. Nevertheless, the present study showed shorter plants with a reduced length of axillary branches compared to the control, but not as much as plants which were only treated with NAA.

Plant architecture was influenced using PGR. The aim was to modify the plant morphology in order to generate small, compact plants for various indoor growing systems. While NAA-treated plants showed a short habitus, including significant the shortest axillary side branches with a reduced number of internodes. BAP-treated plants and plants treated with the NAA/BAP-mix also showed shorter habitus and demonstrated shorter axillary side branches with reduced numbers of internodes. It is important to note that the use of PGR did not reduce biomass yield and the content of cannabinoids. Results showed that the impact of PGR on yield of inflorescence was dependent on the interaction between the genotype and the treatment. NAA- and BAP-treated plants of genotype KANADA showed an equally high yield of inflorescence DW than the control plants, whereas 0.2x genotype showed an equally high DW yield, but only for BAP-treated plants. However, genotype FED indicated a lower DW yield of inflorescence in PGR treatments. It is important to mention that *Cannabis* genotypes respond differently to production conditions, as reported by Backer et al. [45]. CBD contents in inflorescence and leaves showed no impact on PGR. Considering that a minimal range of variation is aimed for and a THC content of < 0.2% must not be exceeded, the fact that PGR applications have no influence on cannabinoid content may be advantageous.

Above all, the inflorescence yields are decisive, since a higher content of cannabinoids is expected. Stout et al. [46] reported the highest CBD levels in *Cannabis* flowers, with lower amounts in leaves. Cannabinoids can be extracted from the reproductive plant parts and foliage. Inflorescence has higher concentrations of cannabinoids than foliage material, however foliage parts comprise the lager biomass of the *Cannabis* plant [47]. The PCR genetics used in the present study showed lower leaf DW compared to inflorescence DW. Leaf DW yield depended on the genotype and treatment, where genotype KANADA showed the highest DW leaf yields and genotypes 0.2x and FED were not that profitable. The use of PGR did not reduce leaf yield for BAP-treated and NAA/BAP-mix-treated plants.

In summary, the results showed that the used PCR genetics reacted differently to PGR applications. Genotype KANADA was shown to be suitable as a treatment with synthetic auxin (NAA) to adapt plant architecture to corresponding indoor conditions. Short plant height was characterized by stability in the generative phase and avoided breakdown of side branches. A defined number of axillary side branches by apical bud removal and a reduced side-branch length guaranteed homogeneous flower development by uniform exposure. Furthermore, better harvesting conditions were given due to uniform plant height and homogeneous plant development. Despite a more compact habitus, the flower yield was not reduced and the KANADA genotype showed the highest CBD content compared to the other genotypes. Treatment with synthetic CK (BAP) was shown to be disadvantageous regarding the PCR genetics used, because axillary side-branch length could not be significantly shortened. Treatment with a mix of both showed no beneficial effects in any of the three genotypes, as the DW of inflorescence was reduced by the treatment. Only leaf DW was not reduced. If leaf yield is the purpose of use, BAP treatment may be appropriate. In this case, genotype KANADA is also recommended due to the significant finding of the highest leaf DW.

The use of specific PGR is permitted in fruit growing, horticulture, and field-crop cultivation [17,48,49]. In the cultivation of medicinal grade *Cannabis* production, there are currently no approved PGR to modify plant architecture. Since the use of PGR in production under good manufacturing practice (GMP) or good agricultural and collection practice (GACP) guidelines is not clearly defined, the use of PGR in the cultivation of nonmedical *Cannabis* could be of importance for cosmetics or nutraceuticals, for example. Nevertheless, minimum effective chemical inputs, including fertilizers, growth regulators, pesticides, and herbicides, should be achieved and well documented to secure the marketability of the product [50]. When using synthetic PGR, it is very important to know exactly the appropriate application methods and concentrations to avoid possible residues in the final product, as high concentrations of IAA are toxic. Because of these properties, compounds with auxin-like activities were developed and can be applied as herbicides [51]. Synthetic IAAs are more stable than endogenous IAA, because the compounds show reduced metabolic turnover [52]. Thus, natural phytohormones could be an alternative to synthetic ones.

## 5. Conclusions

The results of this study showed that exogenously applied plant growth regulators (PGR), namely 1-naphthaleneacetic acid (NAA), 6-benzylaminopurine (BAP), and a mixture of both (NAA/BAP-mix), had significant impacts on the plant architecture of *C. sativa*. Phytocannabinoid-rich (PCR) *Cannabis* genetics reacted in a genotype-specific manner to PGR applications. The use of NAA led to a more compact plant architecture with a consistently high inflorescence yield for the genotype KANADA; cannabidiol (CBD) content was not affected. A beneficial effect on the autoflowering genotype (FED) could not be confirmed. Genotypes 0.2x-genetic and FED showed reduced inflorescence yield due to PGR application. The use of PGR opens up a very interesting field and requires further study to test the use of PGR at different concentrations. Although exogenously applied PGR might be a cultural practice in the future, further studies to screen more PCR-genetics on their specific reactions to PGR applications are required to develop new genotype-specific indoor cultivation systems.

## Figures and Tables

**Table 1:** Mean inflorescence dry weight (DW) in g plant^−1^ of genotypes KANADA, 0.2x-genetic, and FED, treated with NAA, BAP, and NAA/BAP-mix and a nontreated control. Results are presented as mean values ± standard error of mean (mean ± SEM). Means of treatments in one column followed by at least one identical lowercase letter are not significantly different, as indicated by the LSD test (α = 0.05). Means of genotypes in one row followed by at least one identical uppercase letter are not significantly different, as indicated by the LSD test (α = 0.05). The *p*-values correspond to global F tests for differences between the levels of the mentioned genotypes, treatments, or their interactions.

Trait	Treatment	Genotype		
		KANADA	0.2x-genetic	FED
Inflorescence DW [g plant^-1^]	Control	24.31 ± 3.06 ^aA^	20.83 ± 3.79 ^aA^	32.10 ± 3.76 ^aA^
	NAA	23.51 ± 3.10 ^abA^	7.61 ± 3.10 ^bB^	5.39 ± 3.10 ^cB^
	BAP	22.97 ± 3.11 ^abA^	15.88 ± 3.11 ^abA^	17.61 ± 3.13 ^bA^
	NAA/BAP-mix	14.79 ± 3.13 ^bAB^	8.57 ± 3.11 ^bB^	19.12 ± 3.80 ^bA^
***p*-values**				
Genotype [G]		0.0041		
Treatment [T]		0.0003		
G × T Interaction		0.0430		

**Table 2:** Mean leaves dry weight (DW) in g plant^−1^ of genotypes KANADA, 0.2x-genetic, and FED and mean leaves dry weight (DW) in g plant^−1^ of genotypes KANADA, 0.2x-genetic, and FED, treated with NAA, BAP, and NAA/BAP-mix and a nontreated control. Results are presented as mean values ± standard error of mean (mean ± SEM). Means of genotypes or treatments in one row followed by at least one identical lowercase letter are not significantly different, as indicated by the LSD test (α = 0.05). The p-values correspond to global F tests for differences between the levels of the mentioned genotypes, treatments, or their interactions.

Trait	Genotype			
Leaves DW [g plant^-1^]	**KANADA**	**0.2x-genetic**		**FED**
	25.14 ± 1.94 ^a^	18.54 ± 2.07 ^b^		17.76 ± 2.21 ^b^
	**Treatment**			
	**Control**	**NAA**	**BAP**	**NAA/BAP-mix**
	25.6 ± 2.60 ^a^	12.43 ± 2.24 ^b^	22.96 ± 2.24 ^a^	20.79 ± 2.43 ^a^
***p*-values**				
Genotype [G]	0.0337			
Treatment [T]	0.0045			
G × T Interaction	0.1914			

**Table 3:** Mean content of cannabidiol (CBD) in mass percent [%_m/m_] of genotypes KANADA, 0.2x-genetic, and FED. CBD was analyzed in inflorescence and leaves. Results are presented as mean values ± standard error of mean (mean ± SEM). Means in one row followed by at least one identical letter are not significantly different as indicated by LSD test (α = 0.05). The p-values correspond to global F tests for differences between the levels of the mentioned genotypes, treatments, or their interactions.

Trait	Genotype		
	KANADA	0.2x-genetic	FED
CBD [%m/m]			
Inflorescence	10.33 ± 0.30 ^a^	7.91 ± 0.30 ^b^	6.34 ± 0.31 ^c^
Leaves	7.03 ± 0.18 ^a^	6.77 ± 0.20 ^a^	5.59 ± 0.28 ^b^
***p*-values Inflorescence**			
Genotype [G]	0.0226		
Treatment [T]	0.4411		
G × T Interactions	0.1072		
***p*-values Leaves**			
Genotype [G]	0.0026		
Treatment [T]	0.8755		
G × T Interactions	0.7891		
